# Interfacial and Bulk Properties of Potato and Faba Protein in Connection with Physical Emulsion Stability at Various pH Values and High Salt Concentrations

**DOI:** 10.3390/foods13233795

**Published:** 2024-11-26

**Authors:** Jiarui Cao, Meinou Corstens, Karin Schroën

**Affiliations:** Laboratory of Food Process Engineering, Wageningen University and Research, Bornse Weilanden 9, 6708 WG Wageningen, The Netherlands; jiarui.cao@wur.nl (J.C.); meinou.corstens@wur.nl (M.C.)

**Keywords:** plant protein, bulk viscosity, O/W interface, interfacial rheology, emulsions, physical stability

## Abstract

The protein transition motivates the use of plant proteins, but their application in food emulsions is challenging, especially when high concentrations of oil and salt are needed for formulation and sensory properties. In the present work, we connect the iso-electric point of two potato protein isolates (patatin-rich, POPI-200; protease inhibitor-rich, POPI-300) and a faba protein isolate (FPI) to the behavior in the bulk phase and at the interface, and relate this to the physical stability of 45 wt% oil-in-water (O/W) emulsions in the presence of NaCl at pH 4.0–7.0. In the absence of NaCl, a higher bulk viscosity was found at the iso-electric point (IEP), especially for the FPI. In the presence of NaCl, the viscosity of the POPI-200 solutions was highest, followed by POPI-300, and that of the FPI was lowest, irrespective of the pH. Both POPIs showed faster initial adsorption at the O/W interface in the absence of NaCl, and formed a more elastic layer compared to the FPI. For all proteins, salt addition leads to less elastic films. Interestingly, the interfaces were more elastic at a pH close to the IEP of the protein in the presence of NaCl. Both POPI-stabilized emulsions showed higher stability (smaller size and less oiling off) than the FPI-stabilized emulsions, which makes potato proteins relevant for food emulsion product formulation, even under high salt conditions.

## 1. Introduction

Owing to increasing consumer concerns about the sustainability and ethics of animal breeding, plant-based products have attracted more and more attention, and because of this, the properties of plant-based proteins have also gained a lot of attention, as reviewed in [1,2]. Replacing one protein source with another in food product design is far from trivial, especially when food products have stability challenges. For example, food emulsions experience physical destabilization, such as droplet coalescence and oiling off, depending on the composition of the interface, the bulk stabilizing components, and the processing conditions [3], which may be further aggravated by the use of high amounts of salts. What is important to stress is that the properties of plant protein ingredients are greatly influenced by the processes used for their isolation as extensively reviewed by Loveday, and Day and co-workers [4,5]. Furthermore, components that are co-extracted will sometimes have a very pronounced effect on emulsion stability, as recently pointed out by Munch and co-workers [6], with their effect on both physical and oxidative stability.

Several plant protein concentrates or isolates, such as those from soy, pea, potato, and faba bean, are commercially available and have been used as emulsifiers and/or stabilizers in emulsions [7]. Both intrinsic factors (e.g., amino acid composition), which are related to their nutritional value [5], and process-induced changes in protein structure, e.g., during isolation, determine their technical functionality [5,6], which is often far more complex than that of their animal-based counterparts, of which the properties have been investigated more extensively [2,8]. Detailed knowledge of plant protein behavior in stabilizing emulsions still needs to be collected.

On top of intrinsic factors, environmental conditions (such as pH and ionic strength) play an important role in emulsion stability. The conditions determine the actual charge of the proteins and therefore the interactions between proteins, between proteins and droplets, and between droplets. These interactions affect the viscosity of the continuous phase [9], interface coverage, and interfacial rheology, as well as the flocculation and coalescence of droplets [10,11], thus affecting emulsion stability in a multi-faceted way. In particular, a pH close to the iso-electric point (IEP) of a protein is considered extra challenging due to low solubility [5,12]. Still, several studies have found that plant proteins stabilize emulsions well, despite their low solubility (typically <30% at room temperature) [7,13], which may be indicative of particle stabilization, as well as molecular stabilization. These multi-faceted effects have been limitedly described for plant-based proteins, including the effect of denaturation [14,15,16,17,18], where they have been extensively described for, e.g., milk proteins [19]. The addition of salt reduces the electrostatic repulsion between proteins and emulsion droplets, causing them to aggregate [20,21,22]. Several studies investigated the influence of salt on emulsion stability at limited salt concentrations.

In the current work, we focus on three plant-based proteins, a patatin-rich potato protein isolate (POPI-200), a protease inhibitor-rich potato protein isolate (POPI-300), and a faba protein isolate (FPI). Potato and faba protein isolates are promising sources of plant proteins because they are non-allergenic, have high nutritional value, and exhibit functional attributes, including emulsifying and gelling properties [20,23,24,25,26]. Only limited work has been conducted on these proteins in relation to emulsion stability [20,27,28].

Potato has three main proteins: patatin, protease inhibitors, and other higher-molecular-weight proteins. Patatin is a group of homologous isoform proteins with molecular weights from 40 to 45 kDa and an iso-electric point (IEP) between pH 4.5 and 5.2. Patatin contains randomly distributed charged residues [29] and is rich in branched-chain amino acids (isoleucine, leucine, and valine) as well as aromatic side chains (phenylalanine and tyrosine) [30,31]. With a single free thiol group and no internal disulfide bonds [13], the native structure of patatin is primarily stabilized by hydrophobic interactions. Protease inhibitors are a heterogeneous group of smaller proteins with molecular weights from 5 to 25 kDa and an IEP between 5.1 and 9.0 [20,32,33]. Previous research showed that potato proteins had comparable emulsifying properties to whey proteins [34,35]. However, their application in food products is still much less advanced.

Faba bean protein consists of two globulin storage proteins, 11S (legumin; 300–400 kDa) and 7S (vicilin; 150 ± 2.5 kDa) [26]. The IEP of faba proteins ranges from 3.5 to 4.0. Although faba proteins have been reported to be comparable to soy and lentil protein isolates in terms of emulsifying stability [36], their application in food products is still negligible, primarily due to their poor solubility and functionalities compared to animal proteins [37].

In the current study, we use a challenging emulsion: 45 wt% sunflower oil and 0.32 wt% plant protein, at pH 4.0–7.0, in the absence or presence of NaCl. The investigated pH range includes the iso-electric points of the proteins under study, and the addition of high amounts of salt is also expected to affect various factors related to emulsion stability. Therefore, we consider molecular, bulk, and interfacial properties when characterizing these emulsions. The results obtained are expected to provide useful information for savory emulsion products.

## 2. Materials and Methods

### 2.1. Materials

Potato protein isolates (patatin-rich protein, POPI-200; protease inhibitor-rich protein, POPI-300) and a faba protein isolate (FPI) were provided by Royal Avebe (Veendam, The Netherlands) and Barilla G. e R. Fratelli—Società per Azioni (Parma, Italy), respectively. Regular sunflower oil (Borges Agricultural & industrial edible oils, Tarrega, Spain) was from a local supermarket (Jumbo). The soluble protein concentration was determined using a bicinchoninic acid (BCA) kit (Thermo Scientific, Rockford, IL, USA). 8-Anilinonaphthalene-1-sulfonic acid ammonium salt (98%) was purchased from Thermo Fisher Scientific (Waltham, MA, USA). Sodium phosphate dibasic, sodium phosphate monobasic, sodium chloride (NaCl), phosphoric acid, and sodium dodecyl sulfate (SDS) were purchased from Sigma Aldrich (Saint Louis, MO, USA) and were of at least analytical grade. Sodium hydroxide and hydrochloric acid (37%) were purchased from Supelco (EMD Millipore Corporation, Burlington, MA, USA) and VWR Chemicals BDH^®^ (Fontenay-sous-Bois, France), respectively. All chemicals were used as received. Deionized water was obtained from a Milli-Q system (Millipore Corporation, Billerica, MA, USA) and used for all experiments.

### 2.2. Protein Characterization

#### 2.2.1. Differential Scanning Calorimetry (DSC)

The denatured state and thermal properties (denaturation temperature (*T_d_*) and enthalpy change (Δ*H*)) of the plant proteins were determined by differential scanning calorimetry (DSC-250, Mettler Toledo, Schwerzenbach, Switzerland). Protein powders were dispersed in deionized water. Around 68 mg protein solution (25 wt% protein) was loaded into an aluminum cup. After equilibration at 20 °C, the samples were heated from 20 to 150 °C at a rate of 2 °C/min, and then cooled down to 20 °C at the same rate. The process was repeated with an intermittent isothermal phase of 1 min. The heat flow was measured as a function of temperature, and the enthalpy of denaturation (Δ*H*, J/g) was computed from the thermograms using specific software (Universal Analysis Program, Version 2.5H, TA Instruments, New Castle, DE, USA, Waters LLC., Milford, MA, USA).

#### 2.2.2. Zeta Potential and Particle Size Measurements

The proteins were dissolved in distilled water at a concentration of 0.32 wt%. The zeta potential and average sizes were measured by dynamic light scattering (Zetasizer Ultra, Malvern Panalytical Ltd., Almelo, The Netherlands). The refractive indices were set at 1.360 for the POPIs and 1.4506 for the FPI [38,39]. The refractive index of water was taken as 1.330. The pH was varied from 2 to 8 using an MPT-2 titrator (Zetasizer Ultra, Malvern Panalytical Ltd., Almelo, The Netherlands). Three measurements were taken per pH and analyzed with ZS Xplorer 3.0.0.53.

#### 2.2.3. Protein Solubility

To measure solubility, proteins were dispersed in distilled water at a concentration of 0.32 wt%. After stirring for 1 h, the pH was adjusted to values between 2 and 8 using either 1 M HCl or 1 M NaOH. Next, the solutions were put in a rotator at 40 rpm for 30 min and stored in the fridge (4 °C) overnight. The protein solutions were centrifuged at 16,000× *g* for 30 min the next day, after which the protein content of the supernatant was determined using the BCA assay [39], from which the solubility was compared with the amount used to make the solution.

#### 2.2.4. Surface Hydrophobicity

Using 1-anilino-8-naphathalenesulfonate (ANS) as a fluorescent probe, the surface hydrophobicity (*H*_0_) of proteins was measured. The measurement was carried out according to previously described methods [40,41,42]. First, 8 mM ANS and protein solutions with concentrations ranging from 0.01 to 0.2 mg/mL were prepared in 10 mM phosphate buffer (pH 4.0, 5.0, 6.0, 7.0). Next, 20 μL ANS (8 mM) was added to 4 mL of the protein solutions, and the mixtures were stored for 15 min in the dark. After this, the relative fluorescence intensity (RFI) of each sample was measured at excitation and emission wavelengths of 390 nm and 470 nm, respectively, using a Shimadzu RF6000 Fluorimeter (Shimadzu Corporation, Kyoto, Japan). The initial slope of the plot of the RFI vs. protein concentration (mg/mL) was used as an indicator of protein surface hydrophobicity.

### 2.3. Protein Behavior in Bulk and at Oil–Water Interface

Protein solutions (0.58 wt%) were prepared by dispersing protein powders in a 10 mM phosphate buffer at pH 4.0 and 7.0, without and with 5.5 wt% NaCl in the water phase. All solutions were stirred for 2 h at room temperature and stored overnight at 4 °C for complete hydration. The next day, the pH of the protein solutions was readjusted to the desired pH (4.0 or 7.0) using 0.5 M NaOH or 0.5 M HCl before the measurement took place.

#### 2.3.1. Bulk Viscosity of Protein Solutions

The viscosity of the protein solutions was measured using an Anton Paar Rheometer MCR 702 (Anton Paar, Breda, The Netherlands). A concentric cylinder geometry CC17 measuring system with a diameter of 16.66 mm and a length of 24.858 mm was used for 5 mL of each sample. The viscosity was measured across a series of shear rates ranging from 0.01 to 100 s^−1^ at 20 °C.

#### 2.3.2. Oil-in-Water (O/W) Interface Characterization

The TRACKER™ Automatic Drop Tensiometer (ADT) and Dilatational Interfacial Rheometer (Teclis, Civrieux-d’Azergues, France) were used to study adsorption behavior and interfacial rheology in an oil/water system. The protein solutions (0.58 wt%) were diluted using a phosphate buffer of desired pH to reach a final protein concentration of 0.08 g/L.

Interfacial pressure.

The time-dependent interfacial pressure (*π*) was derived from the interfacial tension at the O/W interface of an oil droplet generated by a J-shaped syringe submerged in the protein solution. The interfacial tension *γ* was monitored for 3 h at 20 °C. The interfacial pressure (*π*, mN/m) was calculated from the interfacial tension using the following equation:

(1) $$\pi =\gamma_{0}-\gamma_{t}$$

where *γ*_0_ (mN/m) is the interfacial tension of a bare oil–phosphate buffer interface (~27.5 mN/m) and *γ_t_* (mN/m) is that of the interface in time. Ward and Tordai’s (1946) approach was used to quantify initial protein adsorption at the O/W interface [43].

(2) $$\frac{d_{\pi }}{d_{t^{0.5}}}=K_{diff}=2C_{0}RT{\left(\frac{D}{3.14}\right)}^{0.5}$$

where *C*_0_ is the continuous phase concentration, *R* is the gas constant, *T* is the absolute temperature, and *D* is the diffusion coefficient. From the initial slope of *π* versus *t*^1/2^, the diffusion constant *K_diff_* follows, which in turn can be used to calculate the (apparent) diffusion coefficient (*D*).

(3) $$D=3.14\cdot {\left(\frac{K_{diff}}{2C_{0}RT}\right)}^{2}$$

In this study, *K_diff_* and *D* were calculated for the first 9 s of the measurement.

Dilatational rheology.

After 3 h, when the interfacial tension remained rather constant, oscillatory dilatational deformations were applied with a constant frequency of 0.1 Hz. The droplet interface was compressed and expanded in a sinusoidal way, ranging from 5 to 30% deformation. Five deformation cycles were performed, after which five rest cycles were applied before the next (larger) deformation cycle started. The oscillating surface tension signal was analyzed with a Fast Fourier transform, and the intensity and phase of the first harmonic were used to calculate the elasticity and rigidity modulus, respectively. Outside this regime, higher harmonics occur, which can be analyzed by Lissajous plots (surface pressure plotted against the oscillating deformation signal) [44].

### 2.4. Emulsion Preparation and Characterization

#### 2.4.1. Preparation of Emulsions

To simulate food products, O/W emulsions containing 45 wt% sunflower oil were prepared at pH 4.0, 5.0, 6.0, and 7.0. Protein solutions (0.58 wt%) were made in a 10 mM sodium phosphate buffer in the presence of NaCl (5.5 wt%) and stirred for 2 h at room temperature (~20 °C), after which they were stored in the fridge (4 °C) overnight. The next day, the protein solutions were exposed to a high-speed disperser (S18N-19G, Ultra-turrax IKA T18 digital) (IKA-Werke GmbH & Co. KG, Staufen, Germany) at 3000 rpm for 90 s, after which the sunflower oil was added during mixing at 6000 rpm. A coarse premix emulsion was obtained by increasing mixing to 12,000 rpm for 40 s, after which the pH was adjusted to 4.0, 5.0, 6.0, and 7.0, and homogenization took place using a lab-scale colloid mill (IKA Magic Lab, Staufen, Germany) with a gap width of 0.32 mm at 17,000 rpm for 4 min.

#### 2.4.2. Droplet Size

The droplet size distribution was measured by static light scattering using a Mastersizer 3000 (Malvern Instruments Ltd.; Worcestershire, UK). The refractive index was 1.465 for the dispersed phase (sunflower oil) and 1.330 for the dispersant (water). An absorption index of 0.01 was applied. Emulsions that showed a bimodal distribution were diluted 10 times in the 1 wt% SDS solution prior to measurement to break down possible aggregates and distinguish coalescence. Droplet size was reported as the Sauter mean diameter (*d*_3,2_) and as the average of at least two independent samples, each of which were measured three times.

#### 2.4.3. Protein Surface Load

The cream phase of the emulsion was separated from the continuous phase by centrifugation at 100× *g* for 1 min. Next, the serum phase was centrifuged at 10,000× *g* for 15 min to separate the serum phase and sediment. Then, the serum phase was collected by cautiously making a hole at the bottom of the tube. The soluble protein content was determined with the BCA assay [39]. The sediment at the bottom of tube was dried with nitrogen and the weight of the sediment was measured by using a balance. The surface load *Γ* was calculated with Equation (4).

(4) $$\Gamma =\frac{C_{S}\cdot d_{3,2}}{6\varphi }=\frac{\left(m_{0}-m_{serum}-m_{sediment}\cdot d_{3,2}\right)}{6\varphi \cdot V_{e}}$$

Here, *C_s_* is the adsorbed amount of protein calculated by subtracting the mass of protein in the serum phase (*m_serum_*) and in the sediment (*m_sediment_*) from the mass of protein in the solution used for emulsion preparation, *m*_0,_ and dividing it by the volume used (*V_e_*). In this equation, *d*_3,2_ is the surface weighted mean droplet diameter obtained after dilution with 1 wt% SDS, and *φ* is the dispersed-phase volume fraction.

#### 2.4.4. Gravitational Separation of Emulsion Droplets

Stokes’ law (e.g., Walstra, 2001, [45]) was used to obtain an indication of the gravitational creaming rate (*v*) of ‘freely moving’ droplets:

(5) $$v=\frac{2(\rho_{P}-\rho_{f})}{9\eta }gr^{2}$$

where *g* is the gravitational acceleration constant, *r* is the droplet radius, *ρ_p_* and *ρ_f_* are the density of the sunflower oil and the water phase, respectively, and *η* is the viscosity of the bulk phase [45].

#### 2.4.5. Emulsion Morphology

The droplets were inspected at 40× magnification, using a Carl Zeiss Axioscope A1 microscope equipped with a AxioCam Mrc5 camera (Carl Zeiss Microscopy GmbH, Oberkochen, Germany). The emulsion samples were diluted 5 times in phosphate buffer with the same pH as the emulsion.

### 2.5. Statistical Analysis

All measurements were performed using at least independent duplicates, and means and standard deviations were calculated from these replicates.

## 3. Results and Discussion

### 3.1. Characterization of Plant Protein Isolates

**Figure 1:**
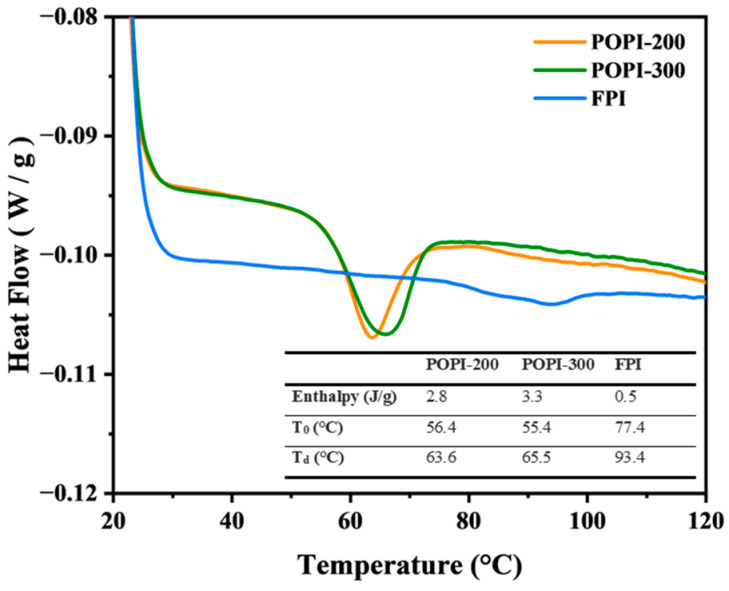
DSC profiles of POPI-200, POPI-300, and FPI. POPI: potato protein isolate; FPI: faba protein isolate.

The potato protein isolates (POPI-200 and POPI-300) showed an endothermic DSC peak, which was hardly visible for the faba protein isolate (FPI). This suggests that the FPI was highly denatured, unlike the POPIs (Figure 1). The related enthalpy was 2.8 J/g for the patatin-rich POPI 200 and 3.3 J/g for the protease inhibitor-rich POPI-300, in line with expectations [20]. POPI-200 and POPI-300 had denaturation temperatures (*T_d_*) of 63.6 and 65.5 °C, respectively, similar to values reported in the literature [3,7,13,27]. The *T_d_* for the FPI was higher (93.4 °C) and similar to a value reported by Hall and Moraru (2021) [46].

**Figure 2:**
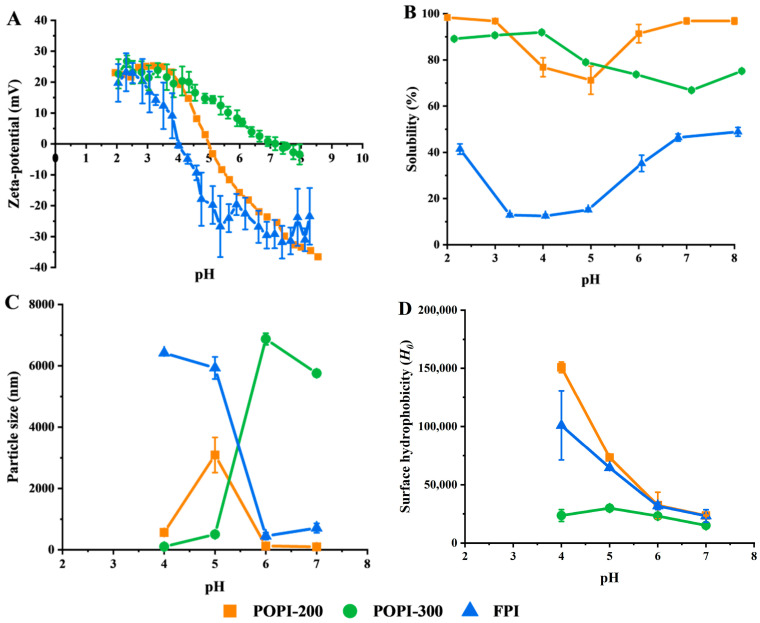
Zeta potential (**A**), solubility (**B**), particle size (**C**), and surface hydrophobicity (**D**) of POPI-200, POPI-300, and FPI at different pH values (10 mM phosphate buffer without additional NaCl).

The iso-electric point (IEP) of the FPI, POPI-200, and POPI-300 was 3.9, 5.0, and 7.2, respectively (Figure 2A). Solubility and particle size (Figure 2B,C) were a function of pH, and as expected, at the respective iso-electric points, massive aggregation took place, as reported by Gumus [47] and reviewed for other proteins [5,12]. The FPI had overall low solubility (12–49 wt%, depending on pH) (Figure 2B) (as reported by Tan et al., 2023) [20]. Surface hydrophobicity is indicative of the amount of hydrophobic residues exposed to the environment [48] and was highest for POPI-200, followed by the FPI and POPI-300 over the investigated pH range (Figure 2D). Hydrophobicity greatly decreased at a high pH. The higher surface hydrophobicity of POPI-200 (patatin-rich protein) is most probably the result of protein unfolding, which can be caused by the low amount of disulfide bonds that are insufficient to keep the protein structure together [49].

When comparing surface hydrophobicity with solubility, it is clear that there is no simple connection. POPI-300 has low surface hydrophobicity and is soluble, and the FPI has high hydrophobicity and is not that soluble, which is as expected, but POPI-200 has high surface hydrophobicity but also has the highest solubility.

To distinguish between effects in the bulk continuous phase (e.g., aggregation, viscosity) and the interface (both can lead to enhanced emulsion stability), the viscosity of protein solutions was investigated first. After that, the interfacial behavior of the proteins was described.

### 3.2. Bulk Viscosity of Protein Solutions

**Figure 3:**
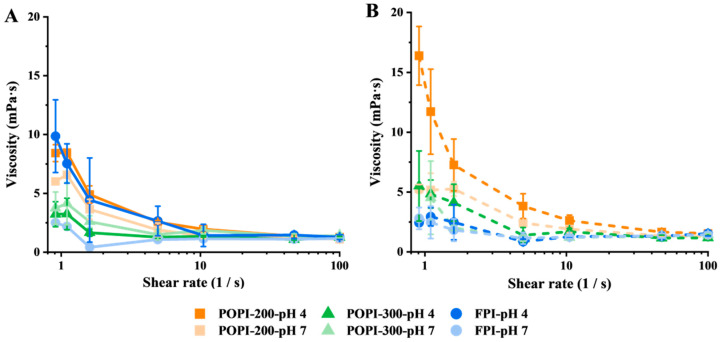
Viscosity as a function of the applied shear rate for the POPI-200 (orange), POPI-300 (green), and FPI (blue) solutions at pH 4.0 (dark color) and pH 7.0 (light color) in (**A**) a 10 mM phosphate buffer and (**B**) a buffer with NaCl.

Figure 3 shows that in the absence of salt, the viscosity of the FPI and POPI-200 is similar at pH 4.0 (~9.9 and 8.4 mPa·s), while POPI-300 shows a significantly lower viscosity (3.2 mPa·s). At pH 7.0, the POPI has a similar viscosity as at pH 4.0, while that of the FPI drops to 2.5 mPa·s (Figure 3A). In the presence of salt, the viscosity of the POPI-200 solutions was highest, followed by POPI-300, and that of the FPI was lowest, irrespective of the pH (Figure 3B).

A direct comparison of the proteins under various conditions can be found in Figure S1. Salt addition at pH 4 increased the viscosity of both potato protein solutions, which we relate to proteins interacting more closely and forming weak networks or aggregated networks. The low viscosity of the FPI (pH 4) in the presence of salt is probably caused by salting-in effects of the globulins, which are soluble under these conditions [50]. At pH 7, the viscosity of all protein solutions with salt was low.

Proteins or other components present in the bulk may lead to a higher viscosity that reduces droplet mobility, or a self-supporting emulsion gel may even form; generally, both are linked to improved emulsion stability [51,52]. Insoluble aggregates may act as inactive fillers [53,54], which could be underlying the viscosity differences found.

### 3.3. Interface Characterization

#### 3.3.1. Adsorption Behavior at the Oil-in-Water Interface

**Figure 4:**
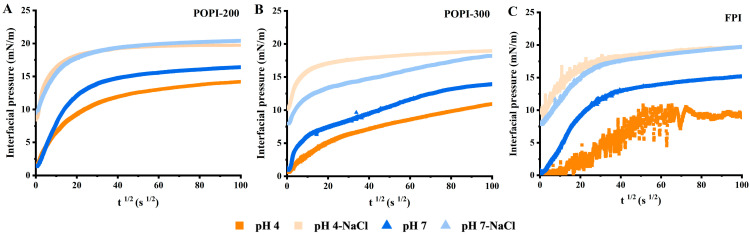
The time dependence of interfacial pressure (*π*) for the POPI-200-(**A**), POPI-300-(**B**) and FPI-(**C**) adsorbed films at the oil–water interface at pH 4.0 (orange) and pH 7.0 (blue) in the absence (dark color) and presence (light color) of NaCl.

Time-dependent interfacial pressure (*π*) measurements at the O/W interface were carried out at pH 4.0 and 7.0. The interfacial pressure increased rapidly and subsequently leveled off for all proteins (Figure 4), as expected for protein adsorption [55]. Steeper increases in interfacial pressure were observed in the presence of salt, and the absolute values after a longer time were higher. Also, the increase in interfacial pressure was higher at pH 4 compared to pH 7, irrespective of the protein used, in the presence of NaCl, while this effect was reversed in the absence of salt. Similar findings were described by Sarigiannidou et al. [17] for pea protein at various NaCl concentrations (0–0.4 M). They ascribed this to counter-ion screening and an increase in protein hydrophobicity [56,57]. There are, however, also alternative interpretations that we elaborate on next.

**Figure 5:**
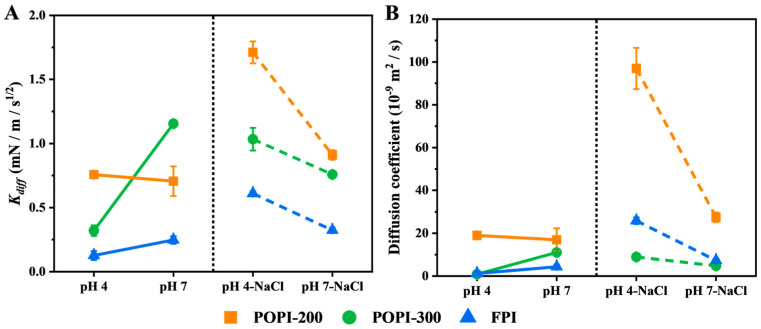
The effect of pH on the protein adsorption rate *K_diff_* (**A**) and apparent diffusion coefficient *D* (**B**) of POPI-200 (orange), POPI-300 (green), and the FPI (blue) at the O/W interface as determined by using the Ward and Tordai model.

We used the modified Ward and Tordai’s equation to quantify interfacial adsorption effects. Depending on pH and NaCl, the initial slope (*K_diff_*; determined within 9 s) varied (see Figure 5A). For the protein solutions without NaCl, both POPI solutions had a relatively higher value of *K_diff_* compared to the FPI, indicating that components present in the POPIs adsorbed at the interface quickly. The lower *K_diff_* values of the FPI near the IEP (pH 4.0) were most probably a reflection of the low solubility (Figure 2B) (and thus lower concentration) and large size (Figure 2C). In the presence of NaCl, *K_diff_* was always higher at pH 4.0 compared to pH 7.0. The values were overall higher for POPI-200 and the FPI than in the absence of salt, while for POPI-300, the *K_diff_* values were in a similar range.

When recalculating the *K_diff_* values to apparent *D* values (Figure 5B) using the following molecular weights, POPI-200: ~40 kDa, POPI-300: ~20 kDa, and FPI: ~58 kDa for subunits, we found high values compared to what would be expected for free diffusion in water (order of 10^−10^ m^2^/s), as also reported by others [55]. This may imply that low-molecular-weight components are present or that certain adsorption aspects are not covered sufficiently by Equations (2) and (3), as is still debated in the literature where many extensions to the original approach can be found (e.g., Felix et al., 2019) [55]. The particle size reported in Figure 2C does not comply with the trends in *K_diff_* measured, clearly indicating that smaller components cause these effects.

#### 3.3.2. Dilatational Rheology

**Figure 6:**
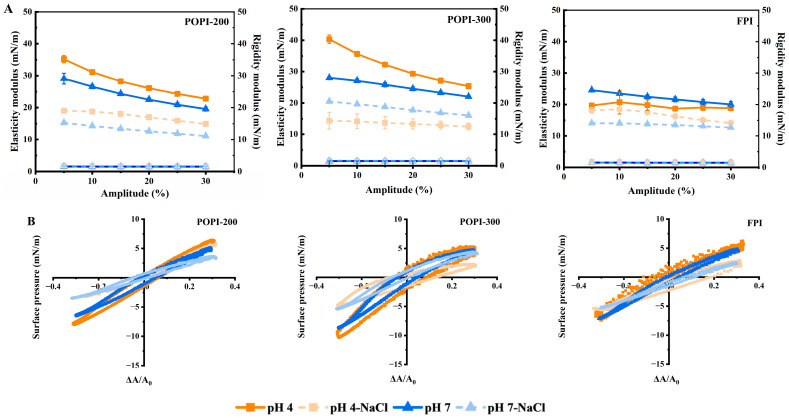
Elasticity (solid) and rigidity moduli (open) (**A**) and Lissajous plots at 30% dilatational deformation (**B**) of O/W interfaces stabilized by POPI-200, POPI-300, and FPI at pH 4.0 (orange) and pH 7.0 (blue) in absence (dark color) and presence (light color) of NaCl.

To understand the protein interactions at the O/W interface, we employed interfacial dilatational rheology. As shown in Figure 6A, the elastic moduli were always higher than the rigidity moduli of the protein-stabilized O/W interfaces, which is indicative of a gel-like elastic protein film formation [55,58].

For the protein solutions without NaCl, the interfaces stabilized by POPI-300 had the highest elastic modulus, followed by POPI-200, and the FPI had the lowest elastic modulus. The elastic modulus decreased with increasing deformation (Figure 6A), an effect that was strongest for POPI-300, followed by POPI-200 and the FPI. The results indicated that the interactions between POPI clusters started to break at a larger amplitude, while the FPI-stabilized interfaces showed weak in-plane interactions of the protein structures. Both POPI-200 and POPI-300 showed a higher elastic modulus at pH 4.0, while for the FPI, the value was higher at pH 7.0, indicative of the formation of more elastic layers at pH values away from the IEP.

The addition of NaCl lowered the elastic modulus (Figure 6A), as was found by others who reported that protein aggregates inhibited the formation of a strong viscoelastic layer at the O/W interface [17,57]. It seems that O/W interfaces in the presence of NaCl are more elastic at a pH close to the IEP of the protein, so POPI-200 at pH 4.0, POPI-300 at pH 7.0, and the FPI at pH 4.0, albeit less elastic than in the absence of salt.

When looking in greater detail at the Lissajous plots (Figure 6B) underlying the data in Figure 6A, we see an asymmetrically narrow ellipse shape at all amplitudes. Upon extension, the ellipse was slightly wider but became very narrow upon compression for both POPIs, which is indicative of interfacial network jamming. Both POPIs mostly form more elastic layers compared to the FPI probably due to their more native protein structure that allows them to interact with other proteins in the interface. For the three interfaces at pH 7.0, the lower left part of the graph has a pointy shape, which means that upon compression and subsequent extension, a similar response in surface pressure was measured, indicative of weak in-plane attractive interactions. Hinderink and co-workers reported a similar shape for pea protein isolate [59].

### 3.4. Emulsion Characterization

**Figure 7:**
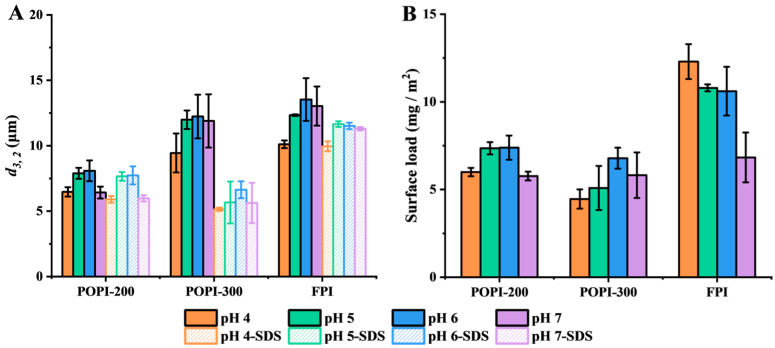
Droplet size (*d*_3,2_) (**A**) and surface load (**B**) of emulsions stabilized by POPI-200, POPI-300 and FPI at pH 4.0 (orange), pH 5.0 (green), pH 6.0 (blue), and pH 7.0 (purple) with addition of NaCl.

The emulsifying ability of the three proteins was investigated through morphology, droplet size distribution, and surface load in O/W emulsions (including 3% NaCl) at pH 4.0–7.0. The emulsions stabilized by POPI-200 and -300 consistently had smaller droplets than those stabilized by the FPI (shown in Figure S2). At a pH close to the IEP (POPI-200 at pH 5.0, POPI-300 at pH 7.2, FPI at pH 3.9), the droplets formed clusters or networks. Earlier studies also reported droplet aggregation at a pH close to the IEP of the used proteins [35,45,47]. Next, we investigated the droplet size and surface load (Figure 7).

In Figure 7A, the droplet size is shown before and after SDS treatment. POPI-200-stabilized emulsions had the smallest droplet size. The droplet size in POPI-200- and FPI-stabilized emulsions remained the same, while POPI-300-stabilized emulsions showed a clear reduction in droplet size upon SDS treatment, suggesting droplet aggregation in the latter emulsion. Similar results were obtained by Van Koningsveld et al. These authors found that patatin-stabilized emulsions did not aggregate, in contrast to protease inhibitor-stabilized emulsions that flocculated, which was also attributed to the presence of polymers [35].

The surface load of the POPI-stabilized emulsions (~6 mg/m^2^) was similar for all pH values, and lower than for the FPI (7–12 mg/m^2^ as a function of pH) (Figure 7B). These values are considerably higher than those reported for small globular proteins such as whey protein isolate (3.2 mg/m^2^), sodium caseinate (3.3 mg/m^2^) [60], bovine serum albumin, α-lactalbumin, and β-lactoglobulin (1–3 mg/m^2^ for monolayer coverage) [61,62,63]. For relatively large globular proteins, such as pea protein isolate [60] and soy proteins [64,65], surface loads of 4–11 and 4.5 mg/m^2^ have been reported, respectively. This can be indicative of interface stabilization taking place by protein aggregates or multilayer formation around oil droplets [47].

### 3.5. Physical Stability and Creaming Rate

Both POPI-stabilized emulsions showed higher physical stability than the FPI-stabilized emulsions. The FPI-stabilized emulsions showed complete creaming within about an hour, while it took about a day for the POPI-stabilized emulsions. This is in line with the calculated creaming rates using Stokes’ law (Figure S3). The POPI-stabilized emulsions had lower creaming rates due to their smaller droplets, and for POPI-200, a higher bulk viscosity was found as well (Figure 3B and Figure 7A). After a five-day storage time, large oil droplets could be observed on top of the FPI-stabilized emulsions, while no oil droplets phase-separated from the POPI-stabilized emulsions (Figure S4).

## 4. Conclusions

We used a multiscale approach to investigate the emulsification behavior of potato protein isolates (POPI-200, POPI-300) and faba protein isolates (FPIs) at various pH values. Emulsion stability is expectedly the result of an interplay among effects taking place: (i) in the bulk, (ii) at the interface, (iii) and between droplets. The low viscosity of the FPI solution in the presence of salt, in conjunction with relatively low surface activity and less elastic interfacial layers, leads to relatively large droplets with low stability. The high surface activity of the POPIs, in combination with the elastic nature of the interfacial layers, leads to emulsions that do not coalesce, not even in the presence of salt, in spite of the tendency of the droplets to flocculate when using POPI-300. These effects are to some extent a function of pH, with POPI-stabilized emulsions being stable at all pHs, while FPI-stabilized emulsions were most stable at the iso-electric point (pH 4.0). In general, POPIs exhibit great potential to formulate stable food-grade emulsions even in the presence of a high amount of NaCl. For FPIs with low solubility and relatively large dimensions, the environmental conditions must be carefully chosen, and particle stabilization should be seriously considered an alternative stabilization mechanism.

## Data Availability

The original contributions presented in the study are included in the article/supplementary material, further inquiries can be directed to the corresponding author.

## Supplementary Materials

The following supporting information can be downloaded at: https://www.mdpi.com/article/10.3390/foods13233795/s1, Figure S1: Viscosity as a function of the applied shear rate for the POPI-200 (A), POPI-300 (B), and FPI (C) solutions at pH 4.0 (orange) and pH 7.0 (blue) in the absence (dark color) and presence (light color) of NaCl. POPI: potato protein isolate; FPI: faba protein isolate. Figure S2: Light microscopy images of emulsions stabilized by POPI-200, POPI-300, and the FPI at the indicated pH on day 1 (2 h after the emulsion formation). Scale bar = 20 μm. Figure S3: The calculated gravitational creaming rate (*v*) of emulsion droplets stabilized by POPI-200, POPI-300, and the FPI at pH 4.0 (orange color) and pH 7.0 (blue color) with the addition of NaCl. Figure S4: Observations of oil separation on the top of the emulsions stabilized by POPI-200, POPI-300, and the FPI on day 1 and day 5.
