# Supplementary material for: Interfacial and Bulk Properties of Potato and Faba Protein in Connection with Physical Emulsion Stability at Various pH Values and High Salt Concentrations
Source: Foods. 2024 Nov 26;13(23):3795. doi: 10.3390/foods13233795 (PMC11640749; doi:10.3390/foods13233795)
Supplement: Supplementary file 1 [file foods-13-03795-s001.zip › foods-3302593-supplementary.pdf]

foods

MDPI

Supplementary material

Interfacial and Bulk Properties of Potato and Faba Protein in Connection with Physical Emulsion Stability at Various pH Values and High Salt Concentrations

Jiarui Cao, Meinou Corstens and Karin Schroën *

Laboratory of Food Process Engineering, Wageningen University and Research, Bornse Weilanden 9, Wageningen 6708 WG, The Netherlands; jiarui.cao@wur.nl (J.C.); meinou.corstens@wur.nl (M.C.)
* Correspondence: karin.schroen@wur.nl; Tel.: +31 (0)317 483396

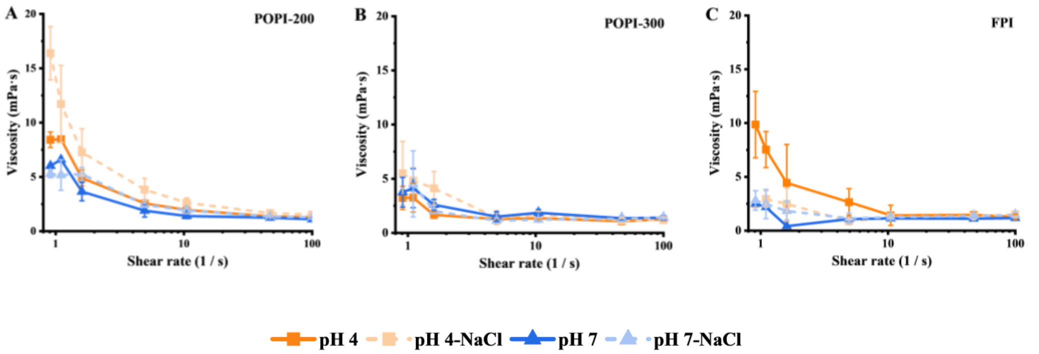

Figure S1. Viscosity as a function of applied shear rate for POPI-200 (A), POPI-300 (B), and FPI (C) solutions at pH 4.0 (orange) and pH 7.0 (blue) in the absence (dark color) and presence (light color) of NaCl. POPI: Potato protein isolate, FPI: Faba protein isolate.

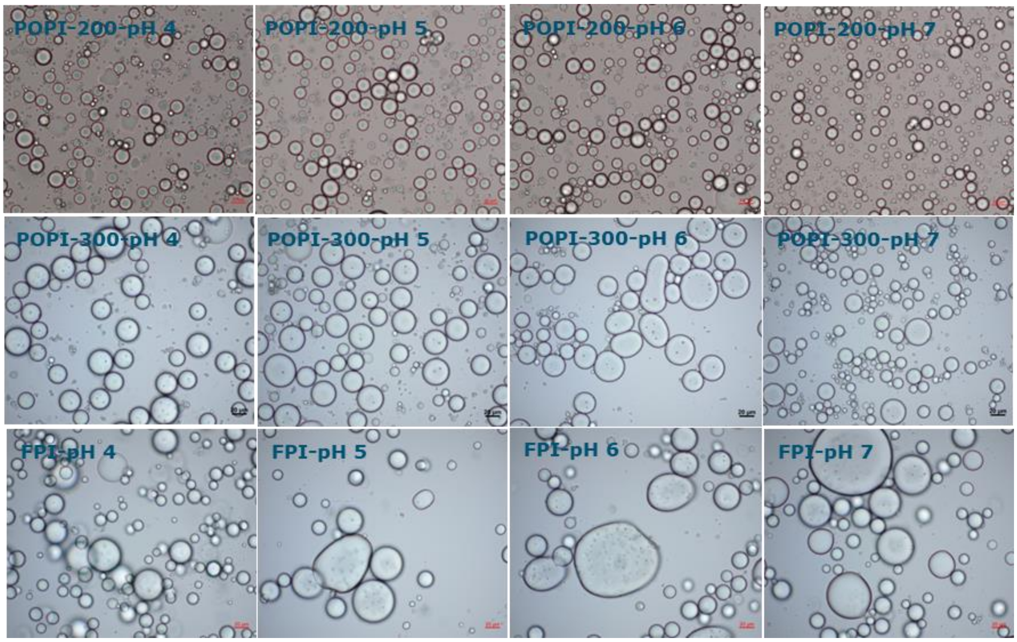

Figure S2. Light microscopy images of emulsions stabilized by POPI-200, POPI-300, and FPI at the indicated pH on Day 1 (2 hours after the emulsion formation). Scale bar = 20 μm.

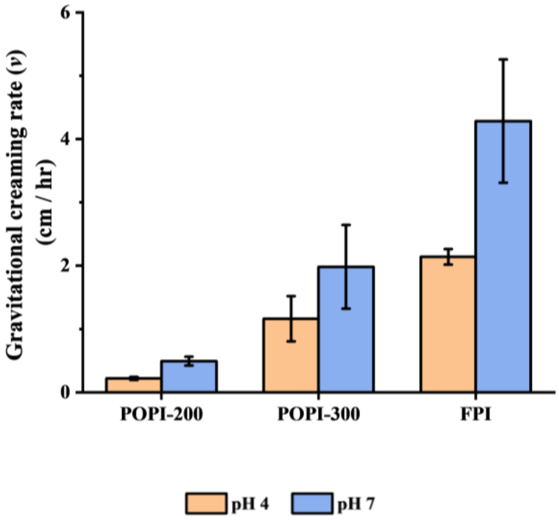

Figure S3. The calculated gravitational creaming rate (*v*) of emulsion droplets stabilized by POPI-200, POPI-300, and FPI at pH 4.0 (orange color) and pH 7.0 (blue color) in addition of NaCl.

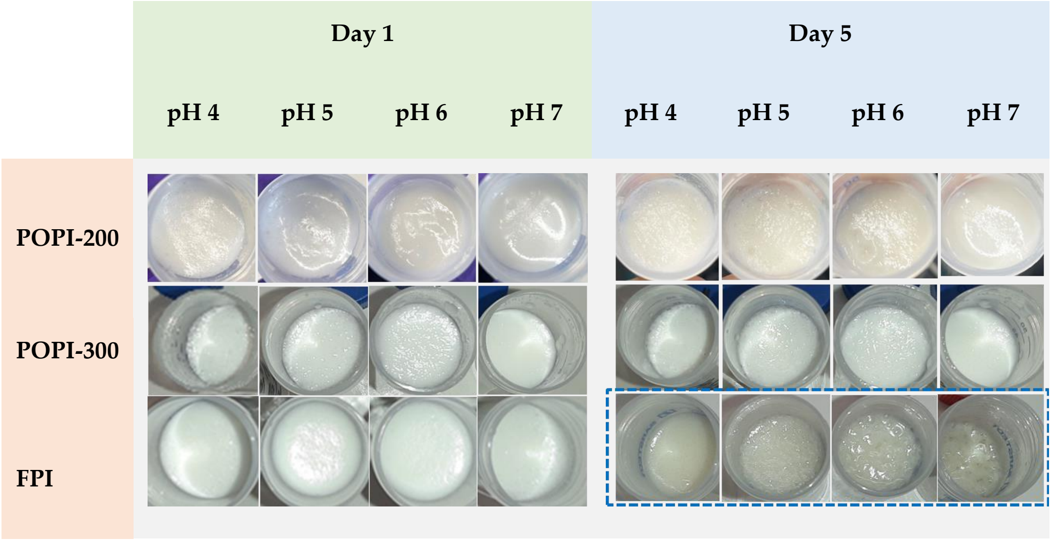

Figure S4. Observation of the oil separation on the top of the emulsions stabilized by POPI-200, POPI-300, and FPI on Day 1 and Day 5.
